# Time to initiate postpartum modern contraceptives among pregnant women in Ambo Town, Central Ethiopia; Cox-proportional hazard regression analysis

**DOI:** 10.1186/s40834-022-00192-x

**Published:** 2022-12-15

**Authors:** Gemechu Gelan Bekele, Ephrem Yohannes Roga, Dajane Negesse Gonfa, Amare Tesfaye Yami

**Affiliations:** Department of Midwifery, College of Medicine and Health Science, Ambo University, Ambo, Ethiopia

**Keywords:** Ambo town, Central Ethiopia, Postpartum modern contraceptive, Survival time

## Abstract

**Background:**

Timing of postpartum family planning is crucial for maternal and child wellbeing by preventing unintended and closely spaced pregnancies. However, studies are limited on the time to use modern contraceptives in Ethiopia. Therefore, this study aimed to fill these gaps by assessing the time to initiate postpartum modern contraceptive and identifying its predictors among pregnant women in Ambo town, central Ethiopia.

**Methods:**

An institution based cross-sectional study was conducted among 356 pregnant women in Ambo town, Central Ethiopia. The data were analysed using STATA-16 software. Kaplan–Meier estimates were performed to explain time-to- modern contraceptive use. A Cox-proportional hazard regression analysis was conducted to identify predictors. The adjusted hazard ratio (AHR) with a 95% confidence interval was considered to declare a statistically significant association.

**Results:**

This study showed that the median survival time to initiate postpartum modern contraceptives was 6 months. In this study, the risk of modern contraceptive use was 2.13 times higher (AHR = 2.13; 95% CI: 1.02–4.45) among younger women, 1.44 times higher (AHR = 1.44; 95% CI: 1.09–2.66) among women with no desire for more children, and 2.25 times higher (AHR = 2.25; 95% CI: 1.02–4.95) among nulliparous women. However, it is 57% times lower (AHR = 0.57; 95% CI: 0.32–0.94) among pregnant women with current unintended pregnancy.

**Conclusion and recommendation:**

The median survival time to initiate postpartum modern contraceptive was 6 months. Age of the women, desire for more children, parity and pregnancy status were found to be the significant predictors of time to initiate postpartum modern contraceptive. Therefore health care providers and concerned stakeholders should consider these factors to increase the uptake of the postpartum contraceptive methods.

## Background

Family planning (FP) is an important part of prenatal, postpartum, and one-year postpartum health care [1]. Globally, the usage of modern contraceptives has increased somewhat, from 54% to 1990 to 57.4% in 2015. In Africa, it increased from 23.6% to 2012 to 28.5% in 2017 [2]. Despite recent gains in boosting the use of contraceptive services, many women of reproductive age still lack access to contraception, resulting in millions of unwanted pregnancies and unsafe abortions each year [3–5]. Preventing unplanned and closely spaced pregnancies significantly reduces mother, baby, and child mortality [6, 7]. Postpartum family planning (PPFP) is crucial in preventing these pregnancies in the first year after delivery [8].

Pregnancy and the postpartum period are considered ideal times for counselling women on adopting a modern family planning method during an extended postpartum period [9].

Ethiopia is Africa’s second-most populous country, after Nigeria, with a total fertility rate of 4.6 children born per woman and a predicted population of 191 million in 2050. Half of the world’s population increase is predicted to be concentrated in only nine nations between 2017 and 2050, including Ethiopia [10, 11]. Despite the fact that modern contraceptives (CPs) have been identified as an effective strategy for fertility regulation, the contraceptive prevalence rate among married women ranged from 5% in Somalia to 53% in Amhara and Addis Ababa. The country’s overall modern contraception prevalence rate is 41%, and 86% of women in the extended postpartum period have an unmet need for family planning [12].

To mitigate this problem and achieve the sustainable development goals (SDG), the World Health Organization (WHO) recommends the integration of FP as an essential component of antenatal care (ANC) and care after birth or abortion. This recommendation includes not only counselling but also documentation of the woman PPFP choice and encouraging her to obtain the method when she returns for a later visit, such as for postnatal care or immunization [13, 14].

Various studies have identified that the timing of postpartum modern contraceptives is associated with maternal age, place of residence, educational status, ANC follow up, exposure to media, average monthly income, place of delivery, and breast feeding status [15–17].

Ethiopia’s government is also committed to achieving the SDGs, and believes that family planning is one of the most important tools for improving maternal health and well-being, and planned to increase contraceptive prevalence rate among married women from 41 to 50% by year the year 2024/25 [18]. However, providers have often neglected this window (ANC contact) to increase demand and actual utilization of PPFP [13].

The majority of previously published articles only assessed the actual contraceptive use during the postpartum period, despite the WHO’s recommendation that pregnant women’s intentions to use postpartum modern contraceptive methods be assessed during ANC follow-up to increase uptake during the postpartum period. Moreover, the earlier research’s use of secondary data sources made them ineffective at identifying the key predictors of initiating postpartum modern contraceptive use. Therefore, this study aimed to fill these gaps by assessing the time to initiate postpartum modern contraceptives and identifying its predictors among pregnant women in Ambo town. The findings of this study could aid planners, programmers, nongovernmental organizations (NGOs), and decision-makers in gaining a better understanding of predictors of contraceptive uptake during the postpartum period. Therefore, increasing postpartum contraceptive uptake plays a significant role in reducing unintended pregnancy rates, which could further decrease maternal, child, infant, neonatal mortality and morbidity rates.

## Methods

### Study design, area and period

An institutional based cross sectional study was carried out in Ambo town public health facilities from November 01-December 01/2021 which are located in West Shewa Zone, Oromia Regional State, Ethiopia. Ambo town is located 114 km away from the capital city of Ethiopia, Addis Ababa. The population of the town is estimated at 64,684 in 2021. The town has two hospitals and two public health centers [19].

## Population

All pregnant women who were attending ANC in public health facilities were considered a source of the population, whereas all pregnant women attending ANC in selected public health facilities in Ambo town during the study period were the study population. Those pregnant women who were attending ANC during the study period and selected with the sampling procedure were study units. All volunteer pregnant women who were available during the study period were included in the study.

## Sample size determination and sampling procedure

The sample size for this study was determined by using a single population proportion formula by considering the following assumptions: 70% of the prevalence of intention on modern contraceptive use among postpartum women in public health institutions of Sodo town, Southern Ethiopia [20], 95% confidence level, 5% marginal error, and 10% nonresponse rate. Based on these, a total of 356 pregnant women were taken as a final sample size.

There are One University Referral Hospital, one General Hospital and two Health centers in Ambo town. Both hospitals were purposively included and One health center namely Awaro health center was randomly selected. The total sample size was divided proportionally to these three public health institutions based on their client flow. The proportionally allocated sample-sized was Ambo University referral Hospital = 160, Ambo General Hospital = 130, and Awaro health center = 66. After reviewing the Antenatal registration book, the average flow of pregnant women attending ANC for two months was taken to determine the sampling interval. Therefore, the average number of pregnant women attending ANC was 1,042 from all sites and yields the sampling interval of three. After selecting the first study participant by using the lottery method, we used a systematic sampling technique to recruit every *k*th eligible respondents from the list of ANC registration serial numbers until the final sample size was reached through the proportional allocation of each clinic.

## Operational definitions

Time: intended time to initiate PPMC and Event: Intention to utilize postpartum PPMC.

Intention to use postpartum modern contraceptive methods is when a woman reported her desire to use any modern contraception methods (pill, intrauterine device, injectable, condom (men or women), sterilization (men or women), or implants) during the 12 months following delivery of this pregnancy [20].

## Data collection tool, quality, and procedures

A structured face-to-face interviewer-administered questionnaire was used to collect the data. The tool was prepared first in English and translated to the local language (Afaan Oromoo) and then translated back to the English language to keep internal consistency. The tool was adopted from a similar study conducted in Ethiopia [17, 20–22]. The questioner contains socio-demographic characters, knowledge of modern contraceptive, maternal and reproductive health characteristics, and intention of future contraceptive use questions. Five bachelor Midwifery collected the data and supervised by one master’s holder in Public health. One day training was provided to both data collectors and supervisor. A pre-test was conducted on 18 pregnant women at Guder health center. Data were checked for completeness and consistency by the supervisors and principal investigator.

## Data processing and analysis

The filled questionnaires were checked for completeness and entered into Epi-Data version 3.1 and exported to STATA version 16 for final analysis. Descriptive statistics were done and presented in the form of tables, figures and texts. Kaplan-Meier survival curves were used to describe the time-to-contraceptive use. Before running the Cox Proportional hazard regression model, the proportional hazard assumption was checked using global goodness of fit based on Schoenfeld residual, and variables having a *P*-value > 0.05 were considered as fulfilling the assumption. The *p*-value for the global test was 0.662, and each predictor variable had a *p*-value greater than 0.05.

To identify potential predictors of time to initiate postpartum modern contraceptive bi-variate Cox proportional regression model was fitted for each explanatory variable. Variables with *p*-value ≤ 0.2 in the bivariate analysis were included in, multivariable Cox proportional hazard regression model. Adjusted Hazard ratio (AHR) with 95% confidence interval (CI) was calculated to estimate the strength of association between independent predictors and time to initiate postpartum modern contraceptive. *P* value ≤ 0.05 was considered for statistical significance.

## Results

### Socio-demographic characteristics

A total of 356 pregnant women participated in the study making a response rate of 100%. The mean age of the study participants was 28 years with standard deviation (SD) of 4.7 years. Majority of the study participants were married 354 (99.4%), Oromo by ethnicity 344 (96.6%), Occupation of house wife 133 (37.4%) and 124 (34.8%) of them were attended college level and above. Regarding their husbands, 217 (61%) of them had attended college and above level and 199 (55.9%) of them stated their occupation is government employee (Table 1).

**Table 1:** Socio-demographic characteristics among pregnant women in Ambo town, central Ethiopia, 2021 (*N* = 356)

Variables	Category	Frequency	Percentage %
Age	≤25	76	21.3
	26-34	235	66.1
	≥35	45	12.6
Ethnicity	Oromo	344	96.6
	Amhara	12	3.4
Religion	Orthodox	159	44.7
	Muslim	47	13.2
	Protestant	136	38.2
	Wakefata	14	3.9
Educational Status Of Mother	No Formal Education	53	14.9
	Read And Write	103	28.9
	Primary Education	19	5.3
	Secondary Education	57	16.0
	Diploma And More	124	34.8
Education Of Husband	No Formal Education	13	3.7
	Read And Write	46	12.9
	Primary Education	19	5.3
	Secondary Education	61	17.1
	Diploma And More	217	61.0
Mother Occupation	Government Employee	117	32.9
	Merchant	78	21.9
	Farmer	27	7.6
	House Wife	133	37.4
	Daily Labourer	1	.3
Husband Occupation	Government Employee	199	55.9
	Merchant	102	28.7
	Farmer	43	12.1
	Daily Labourer	9	2.5
	Other	3	.8
Monthly Income in Ethiopian Birr^a^	<=1380	36	10.1
	1381-6900	113	31.7
	6901-13800	132	37.1
	13801 and above	75	21.1
Place Of Residence	Urban	306	86.0
	Rural	50	14.0
Distance From Nearby Health Facilities	<5km	187	52.5
	5-10km	118	33.1
	>10km	51	14.3

^a^WHO income classification for developing countries (2018)

## Reproductive and maternal health service use-related characteristics

The majority of the participants were multigravida 263 (73.8%) and 219 (83.2%) of them had ANC follow-up for their last pregnancy. Of the participants who received ANC follow up during their last pregnancy, only 43 (16.3%) counseled about PPFP. Majority 237 (90.1%) of the women gave birth at health facilities and 185 (70.3%) received the postnatal care (PNC) services. Among the participant who received the PNC 84 (45.5%) women counseled about family planning during their PNC follow-up. About two third 173 (65.7%) of the women had a birth interval of 24 or more months. Nearly three fourth 271 (76.1%) of the pregnant women stated that, their partner approve the use of FP and 275(77.2%) reported that they decide with their husband to use Modern contraceptive methods (Table 2).

**Table 2:** Reproductive and maternal health service use-related characteristics among pregnant women in Ambo town, central Ethiopia, 2021 (*N* = 356)

Variables	Category	Frequency	Percentage %
History Of Abortion	No	239	90.8
	Yes	24	9.1
Parity	Nulliparity	93	26.1
	1-4	223	62.6
	>=5 (Grand multipara)	40	11.2
No of Living Children	No child	94	26.3
	1-4	223	62.5
	>=5	40	11.2
Sex Proportion	Male Is More	115	43.7
	Female Is More	65	24.7
	Equal	83	31.5
Want More Children	No	49	18.6
	Yes	214	81.3
	Health facilities	61	23.1
	Hospital	176	66.9
Mode Of Delivery Of Index Child	SVD	201	76.4
	Forceps/Vacuum	44	16.7
	CS	18	6.8

## Knowledge and practice of family planning

Majority 340 (95.5%) of respondent heard at least one FP methods. From those the most known methods was DMPA 312 (91.8%), Implanon 303 (89.2%), Pills 297 (87.4%), Male condom 297 (87.4), IUCD 280 (82.4%). Bilateral Tubal Ligation (BTL) 97 (28.5%), Vasectomy 50 (14.7) and Lactational Amenorrhea Method (LAM) 40 (11.8%) were the least known method by the participants. Two hundred twenty (64.7%) knew that the family planning methods prevents unwanted pregnancy and the about half 168 (49.4%) knew that family planning methods limit the number of children and spacing. Nearly half 167 (46.9%) of the women participated in this study has used modern contraceptive at least once in their life.

## Time to initiate postpartum modern contraceptive methods

**Fig. 1:**
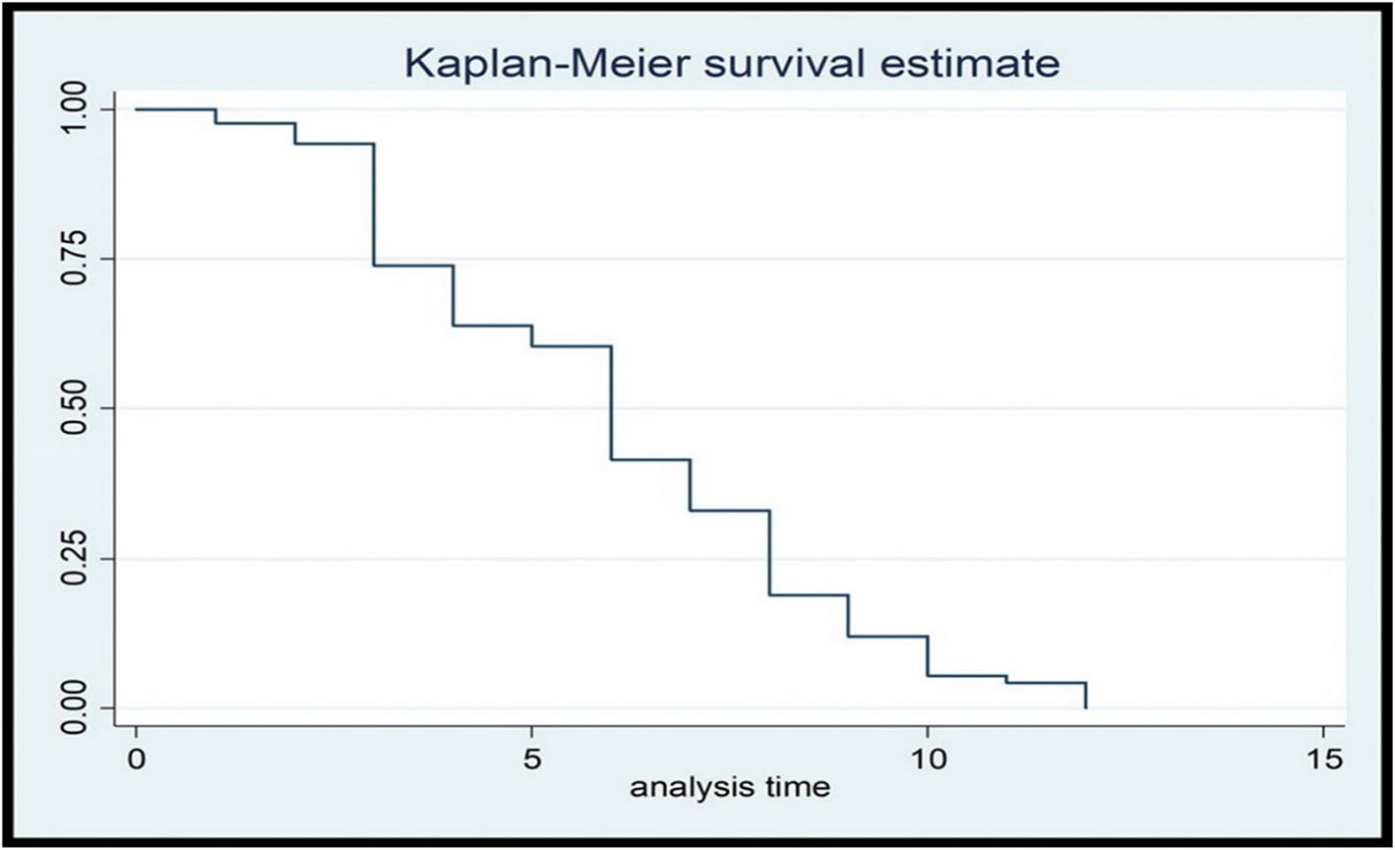
The Kaplan-Meier survival curve for time to initiate Modern Contraceptive methods during the extended postpartum period, Ambo town, Central Ethiopia, 2021

**Fig. 2:**
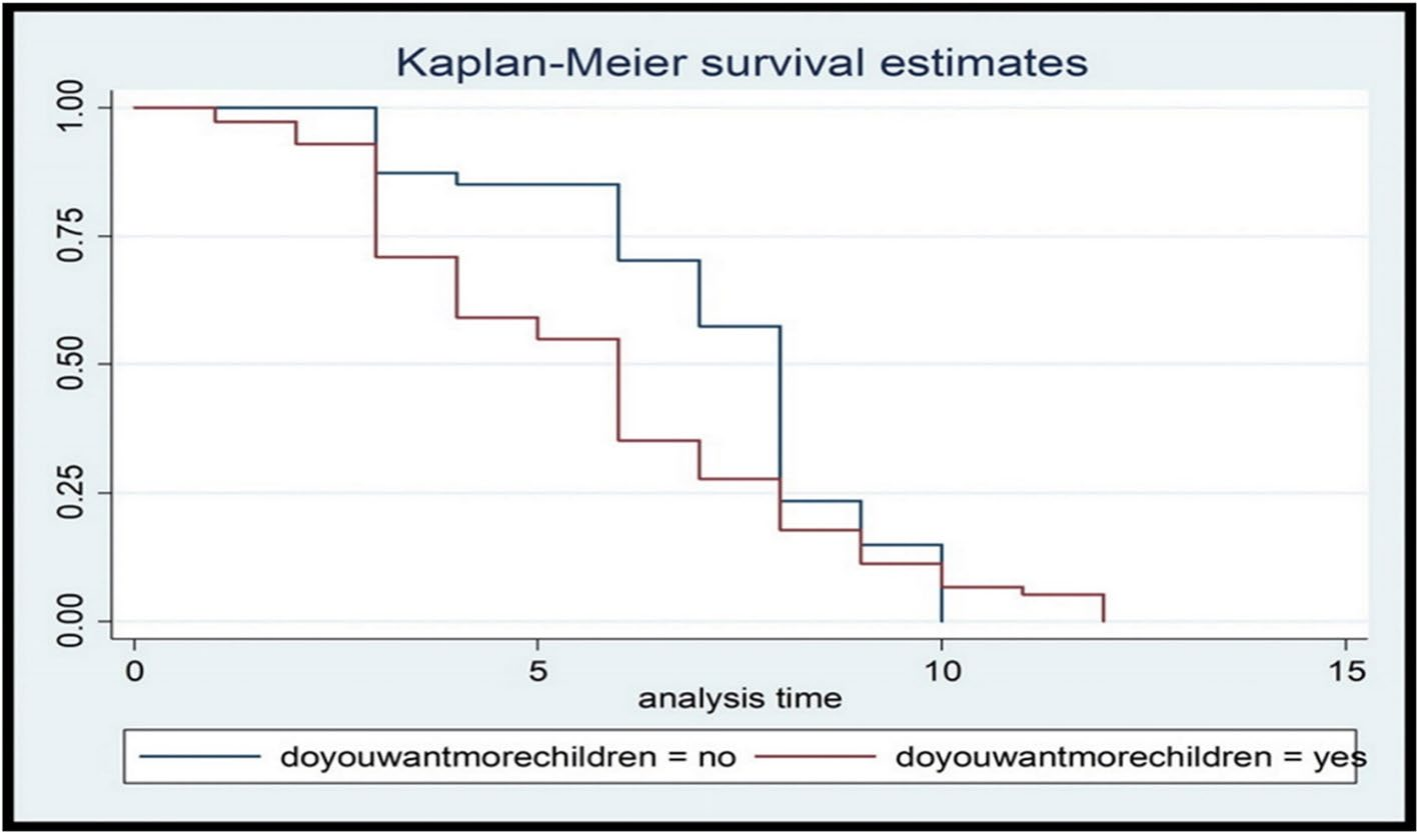
Kaplan Meier graph showing survival time of postpartum modern contraceptive initiation by current pregnancy status among pregnant women in Ambo town, Ethiopia, 2021

**Fig. 3:**
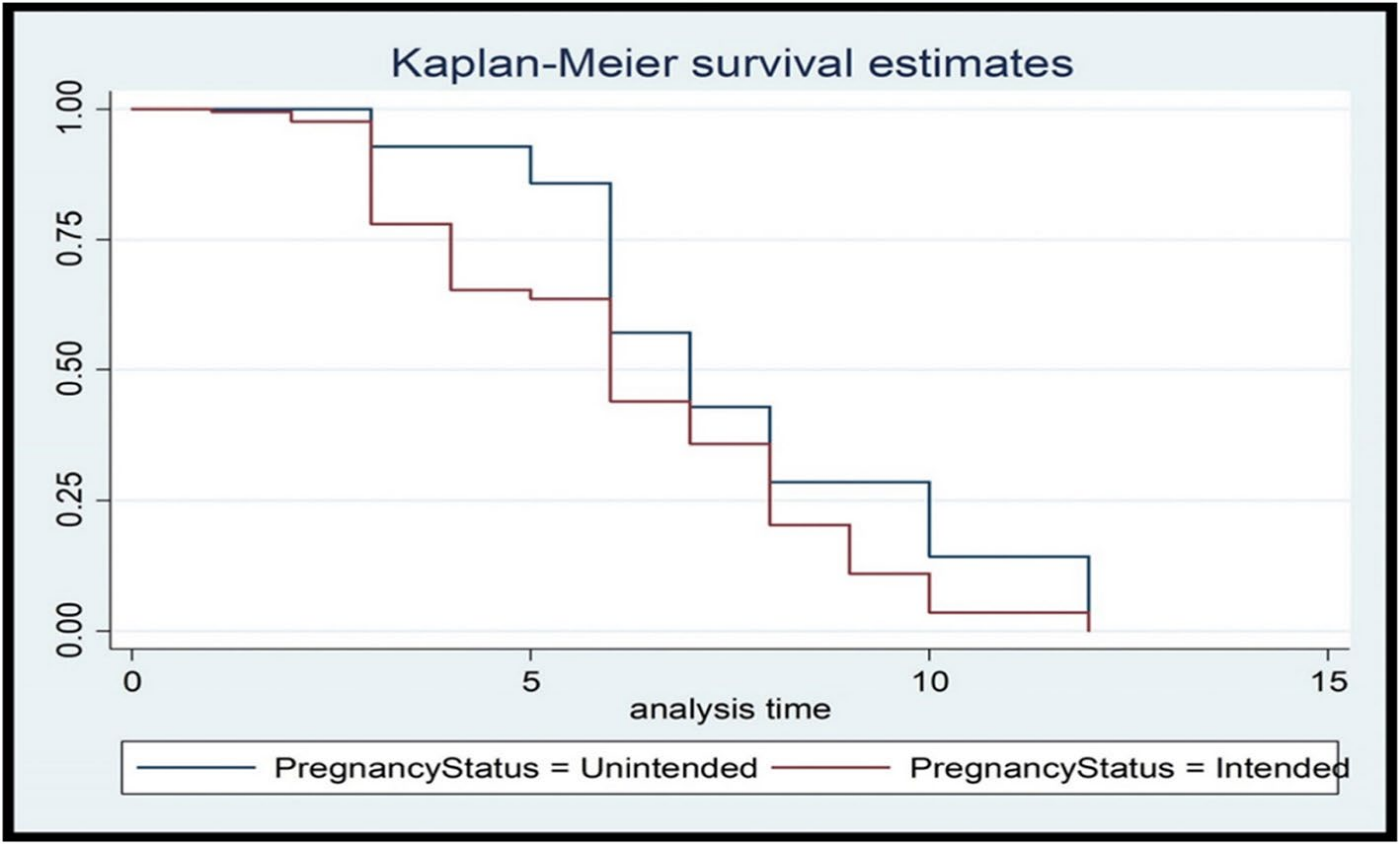
Kaplan Meier graph showing survival time of postpartum modern contraceptive initiation by desire for more children among pregnant women in Ambo town, Ethiopia, 2021

The prevalence of intention to use PPFP was 72.8% (95% CI: 68-76.8). The median time to use modern contraceptive during the extended postpartum period was 6 months with inter quartile range of 5 months (Figs. 1, 2 and 3). From those woman who have intention to use PPMC, above half 148 (57.1%) intended to use implants followed by DMPA 67 (25.8%), IUCD 21 (8.1) COC 15(5.7%) and the least desired method was the POP by 8(3%). The main reason for not intending to use PPMC was desire for more child 33 (34%) and 25(25.7%) described it is sinful or prohibited by religion to use family planning. Fear of side effects 8(8.2%), medical problem 6 (6.1%), and partner disapproval 3(3.0%) were also another reason for not planning to use modern contraceptive within 12 months after delivery.

## Predictors of time to initiate postpartum modern contraceptive methods

Before using the Cox proportional hazard regression model the fitness was checked and the results of the specification test showed that the model was correctly fitted, as predicted by the Hat-statistic (*P* < 0.05).

After controlling for possible confounders Multivariable Cox-proportional hazard regression analysis showed that pregnancy status, parity, and age at marriage were found to be the significant predictors of time to initiate PPMC methods at *p*-value ≤ 0.005.

In this study, the risk of modern contraceptive initiation during the extended postpartum period were 2.13 times higher [AHR = 2.13, 95% CI; (1.02–4.45)] among pregnant women whom their age were less than 25 years, 1.44 times higher [AHR = 1.44, 95% CI; (1.09–2.66)] among women who have no desire for more children, and 2.25 times higher [AHR = 2.25, 95%CI; (1.02–4.95)] among nulliparous women. However it was 0.57 times lower [AHR = 0.57, 95% CI; (0.32–0.94)] among pregnant women whose their current pregnancy were unintended (Table 3).

**Table 3:** Multivariate Cox-proportional hazard regression analysis on predictors of time to initiate postpartum modern contraceptive methods among pregnant women in Ambo town, Central Ethiopia, 2021

Variables	Category	Intention on PPMC		CHR (95% CI)	AHR (95% CI)
		Yes (%)	No (%)		
Age of the participants	≤25	66 (86.8)	10 (13.2)	1.33 (0.91-1.97)	2.13 (1.02-4.45)*
	26-34	168 (71.5)	67 (28.5)	1.31 (0.94-1.84)	1.50 (0.69-3.26)
	≥35	43 (95.6)	2 (4.4)	1	1
Pregnancy status	Intended	186 (75)	62 (25)	1	1
	Unintended	8(57.1)	6 (42.9)	0.66 (0.38-1.14)	0.57 (0.32-0.94)*
ANC follow up	Yes	170 (76.9)	51 (23.1)	1	1
	No	30 (73.2)	11 (26.8)	0.67 (0.45-1.0)	0.83 (0.49-1.43)
Place of delivery	Home	12 (46.2)	14 (53.8)	1	1
	Health facility	188 (79.7)	48 (20.3)	1.56 (0.87-2.82)	0.85 (0.36-2.04)
Received PNC	Yes	144 (77.8)	41 (22.2)	1	1
	No	56 (72.7)	21 (27.3)	0.80 (0.59-1.09)	0.97 (0.67-1.40)
Want more children	Yes	227 (74.9)	76 (25.1)	1	1
	No	50 (94.3)	3 (5.7)	0.75 (0.55-1.03)	1.44 (1.09-2.66)*
Parity	Nulliparous	77 (82.8)	16 (17.2)	1.62 (1.09-2.41)	2.25 (1.02-4.95)*
	Primiparous	164 (73.5)	59 (26.5)	1.42 (0.98-2.04)	1.59 (0.90-2.81)
	Multiparous	36 (92.3)	3 (7.7)	1	1
Place of residence	Rural	45 (86.8)	7 (13.2)	1	1
	Urban	231 (76.2)	72 (23.8)	0.82 (0.63-1.06)	2.05 (1.69-4.03)
Number of children	No child	77 (81.9)	17 (18.1)	1	1
	1-4	164 (73.5)	59 (26.5)	0.88 (0.67-1.45)	1.10 (0.91-1.89)
	≥5	36 (92.3)	3 (7.7)	0.62 (0.41-0.92)	0.65 (0.56-2.12)

*P-value < 0.05 (Statistically significant)

## Discussion

In this study, an attempt has been made to estimate the time to initiate modern contraceptives and its predictors in the first year after delivery. The study revealed that the median time to use modern contraceptives during the extended postpartum period was 6 months with inter quartile range of 5 months. In line with this, the study conducted in Gondar city, Ethiopia and Kenya showed that the median survival time to initiate postpartum modern contraceptives was 6 months [17, 23]. The finding of this study is higher than one previous study conducted in Ethiopia [16]. The variation could be due to the fact that the previous study considered the survival time from the initiation of sexual intercourse after delivery, while this study assessed their intention to use modern contraceptives during the extended postpartum period starting immediately after delivery of the placenta. The result of this study is lower than the study conducted in Uganda, where the median time was 19 months [15]. The possible reason for this variation might be that the study conducted in Uganda assessed time-to-contraceptive use, estimated in months, by the period from the resumption of sexual intercourse following a birth to the time when a woman used modern contraception in the five years preceding the survey. However, this study considered only the 12 months after delivery.

This study showed a significant difference in time to contraceptive initiation among the different age groups. The risk of modern contraceptive initiation during the extended postpartum period was 2.13 times higher among pregnant women whose their age was younger than 25 years. Previous studies conducted in different parts of Ethiopia support this finding [21, 24–26]. This implies that young women are more sexually active than elderly women could explain this [8]. However, the current outcome contrasts with the secondary data analysis of Afghanistan’s 2012 Demographic and Health Survey, which found that women over 40 had a larger demand for modern contraceptive methods [27]. The disparity could be explained by the socio-economic and socio-cultural variations between the countries. As a result, the increased risk of postpartum contraceptive use by women under the age of 25 years may reflect or imply the efforts to prioritize adolescents in pregnancy prevention programs [28] because they are a subpopulation at increased risk for unintended pregnancy [29], as well as short inter pregnancy intervals [30].

Pregnant woman with an unintended current pregnancy had a 57% lower risk of initiating the modern contraceptive during the extended postpartum period compared with those with an intended current pregnancy. The previous study conducted in Ethiopia also revealed that women who had an unplanned pregnancy were less likely to initiate and seek ANC and PNC than women with an intended pregnancy. As a result, pregnant women with current unintended pregnancy were less likely to receive counselling about FP [31]. This was in line with the study conducted in Southern Ethiopia [22, 32, 33] where the higher odds of contraceptive utilization were observed among pregnant women with intended pregnancy. This implies that preventing unintended pregnancy could on the other hand increase utilization of postpartum modern contraceptives utilization.

The results of this study also showed that the risk of postpartum modern contraceptive utilization was 1.44 times higher among pregnant women with no desire for more children. This finding was supported by the results of the study conducted in the Tigray region of Ethiopia [34]. Those participants who have the desire to have more children have a low intention to initiate postpartum modern contraceptives. In this study, 42.6% of Muslims, 9.3% of Protestants and 23.4% of Orthodox have no intention of initiating modern contraceptives during the extended postpartum period. This could be due to the fact that in some religions in Ethiopia, children are seen as a blessing and a gift from the Almighty (God/Allah). As result, it is sinful to prevent pregnancies. In these religions, each sexual act in a marriage needs to be open to the possibility of conceiving a child.

The number of children was also independently associated with the intention to utilize postpartum modern contraceptive methods. This indicated that the risk of postpartum modern contraceptive initiation within the extended postpartum period was 2.25 times higher among nulliparous pregnant women compared with multiparous women. The finding this study is in contrast with the results of with secondary data analysis of Ethiopian (2016) Demographic and Health survey, where woman who had no child and who had 1–2 child/children was nearly 75 and 34% times less likely utilized modern contraceptive method as compared to a woman who had born 5 or more children. This difference might be related with socio-economic status and change of knowledge and attitude of women on contraception over a time.

## Strength and limitation of the study

The study utilized the Cox proportional hazard regression and assessed the time to initiate postpartum modern contraceptive methods and its predictors, which is a great input for policymakers and care providers. However, the study only assessed the intention of pregnant women and didn’t assess the actual utilization time during the extended postpartum period. Therefore, we recommend our readers to consider this limitation while citing and interpreting the findings of this study.

## Conclusion

The study revealed that the median time to use modern contraceptives during the extended postpartum period was 6 months and the prevalence of the intention to use postpartum modern contraceptives was 72.8%. The younger age group, unintended pregnancy, desire for more children, and being nulliparous were significant predictors of time to initiate the modern contraceptives in the extended postpartum period. Health policymakers and healthcare providers should give great emphasis to promote postpartum modern contraceptive method utilization and should consider these factors for its better success.

## Data Availability

Full data for this research is available through the first author up on request.

## Abbreviations

AHR: Adjusted Hazard Ratio
CHR: Crude Hazard Ratio
MC: Modern Contraceptive
PPFP: Postpartum Family Planning
PPMC: Postpartum Modern Contraceptive
